# Assessment of Dorsiflexion Ability across Tasks in Persons with Subacute SCI after Combined Locomotor Training and Transcutaneous Spinal Stimulation

**DOI:** 10.3390/bioengineering10050528

**Published:** 2023-04-26

**Authors:** Jasmine M. Hope, Edelle C. Field-Fote

**Affiliations:** 1 Hulse Spinal Cord Injury Research Laboratory, Crawford Research Institute, Shepherd Center, Atlanta, GA 30309, USA; 2 Neuroscience Graduate Program, Graduate Division of Biological and Biomedical Sciences, Emory University, Atlanta, GA 30322, USA; jhope@emory.edu; 3 Division of Physical Therapy, School of Medicine, Emory University, Atlanta, GA 30322, USA; 4 Program in Applied Physiology, School of Biological Sciences, Georgia Institute of Technology, Atlanta, GA 30318, USA

**Keywords:** foot drop, locomotor training, transcutaneous spinal stimulation, spasticity, SCI

## Abstract

In people with spinal cord injury (SCI), transcutaneous spinal stimulation (TSS) has an immediate effect on the ability to dorsiflex the ankle, but persistent effects are not known. Furthermore, TSS has been associated with improved walking, increased volitional muscle activation, and decreased spasticity when combined with locomotor training (LT). In this study, the persistent impact of combined LT and TSS on dorsiflexion during the swing phase of walking and a volitional task in participants with SCI is determined. Ten participants with subacute motor-incomplete SCI received 2 weeks of LT alone (wash-in phase), followed by 2 weeks of either LT + TSS (TSS at 50 Hz) or LT + TSS_Sham_ (intervention phase). There was no persistent effect of TSS on dorsiflexion during walking and inconsistent effects on the volitional task. There was a strong positive correlation between the dorsiflexor ability for both tasks. There was a moderate effect of 4 weeks of LT on increased dorsiflexion during the task (d = 0.33) and walking (d = 0.34) and a small effect on spasticity (d = −0.2). Combined LT + TSS did not show persistent effects on dorsiflexion ability in people with SCI. Four weeks of locomotor training was associated with increased dorsiflexion across tasks. Improvements in walking observed with TSS may be due to factors other than improved ankle dorsiflexion.

## 1. Introduction

Walking is one of the top rehabilitation priorities after spinal cord injury (SCI) [1]. After SCI, limitations in the ability to achieve adequate ankle dorsiflexion can contribute to impaired ability to achieve foot clearance. This debilitation, known clinically as foot drop, interferes with walking ability and increases fall risk [2]. Since the dorsiflexors (i.e., the tibialis anterior) are the lower extremity muscles most highly influenced by supraspinal input [3,4], damage to the corticospinal tract after SCI has a considerable impact. Although the corticospinal tract descending drive influences dorsiflexion during isolated joint movement and walking [3], spinal pattern generating circuits also strongly contribute to the latter [5,6]. Furthermore, the additional proprioceptive input during weight-bearing stepping compared to isolated joint movement increases the activation of motor neuron pools responsible for dorsiflexion [7]. Taken together, these processes would seem to promote a greater ability to activate dorsiflexors during walking compared to isolated volitional ankle movements.

In addition to impaired ability to activate dorsiflexors, damage to the spinal cord results in other challenges that further complicate ankle control, including the involuntary muscle contractions and stiffness associated with spasticity in the plantar flexors [8]. During walking, involuntary activation of the plantar flexors during the terminal stance may impede the ability to activate the dorsiflexors [9]. Consequently, the presence of plantar-flexor spasticity would seem likely to be associated with lower ability to activate the dorsiflexors during walking, compared to isolated volitional movement. 

Transcutaneous spinal stimulation (TSS) activates the dorsal root fibers carrying afferent information [10,11,12], which influences both volitional and reflex motor output [13,14,15]. Prior studies have provided evidence for the immediate effect of TSS on plantar flexor spasticity and dorsiflexor activation. During 30 Hz frequency of TSS in persons with incomplete SCI, there was an immediate increase in maximum active range of motion (AROM) during a rhythmic ankle task [16]. Additionally, in persons with multiple sclerosis, 50 Hz frequency of TSS improved walking outcomes 2 h post-stimulation and spasticity outcomes both 2 and 24 h post-stimulation [17]. To better understand the temporal persistence of TSS in persons with SCI, there is a need for studies assessing the effects of multiple applications of TSS in conjunction with task-specific training [16]. 

Task-specific training is founded on theories of Hebbian learning [18], wherein specificity and repetition are thought to be essential for improvements in performance [19]. Therefore, to improve locomotor outcomes, therapists have utilized locomotor training (LT) emphasizing repetitive stepping practice to improve gait quality in persons with SCI [20]. Unfortunately, most persons with SCI do not receive the number of repetitions needed to experience the optimal effects of this rehabilitation intervention [21]. To address this challenge, interventions that prime the nervous system have value for enhancing the efficacy of training [22]. Although TSS has been shown to improve walking outcomes, such as speed and endurance [17], the basis for these improvements has not been explored. One possibility is that it contributes to improved ankle control [16], thereby allowing for improved foot clearance. In the development of TSS interventions to improve functional outcomes, it is important to understand how it influences both voluntary and involuntary muscle activation and how it may contribute to the ability to achieve adequate dorsiflexion [23].

Beyond examining the influence of TSS on ankle control, it is important to understand whether there is greater preservation of dorsiflexion as part of the pattern of walking compared to during an isolated, volitional motor task. Although previous studies in persons with SCI have attempted to examine the relationship between volitional activation of the dorsiflexors during a task and walking outcomes [24,25,26], the contribution of ankle dorsiflexors during the swing phase in these improvements has yet to be identified. For instance, in a study that examined the relationship between corticospinal tract descending drive to the tibialis anterior and dorsiflexion during the swing phase, toe clearance and ankle excursion were calculated [24]. However, the clearance can be influenced by hip and knee joint changes, and measurement of ankle excursion during the swing phase can be biased due to increased plantar flexion at the initiation of the swing. To best understand the role of dorsiflexor activation during the swing phase and how it is impacted by noninvasive stimulation, peak dorsiflexion during the swing phase must be analyzed. 

Since the ability to activate the dorsiflexors in isolation may or may not be reflected in the ability to achieve dorsiflexion during walking, it is important to test dorsiflexor control under both conditions. In addition, there is value in understanding the relationship between plantar flexor spasticity and dorsiflexor control. The aim of the current study was to determine whether LT combined with TSS is associated with a persistent effect on dorsiflexion during walking and dorsiflexor AROM in persons with subacute motor-incomplete SCI beyond that observed with LT alone. Additionally, the relationship between plantar flexor spasticity and the ability to dorsiflex across the different tasks (walking and volitional control) was assessed. Due to the role of CPG in walking and because LT is being utilized in this study, we predicted that dorsiflexion during walking would show greater improvements than dorsiflexion during the volitional task.

## 2. Materials and Methods

This study was carried out with ethics approval from the Shepherd Center Research Review Committee. All participants provided written informed consent prior to study enrollment in accordance with the Declaration of Helsinki, and the study was conducted in accordance with the Health Insurance Portability and Accountability Act guidelines. The data used to address the questions of relevance for control of ankle dorsiflexors are a subset of data collected as part of a larger study (see for recruitment and additional screening details) [27], which was registered with clinicaltrials.gov (NCT03240601). 

### 2.1. Participants

Individuals were eligible for participation if they met the following inclusion criteria: 16–65 years of age, 2–6 months since the time of spinal cord injury, qualify for participation in a clinical LT program as determined by their physical therapist, and able to walk 10 m without the use of an ankle–foot orthosis. Individuals were excluded for the following reasons: neurological level of injury at or below T12, progressive or potentially progressive spinal lesions, history of cardiovascular irregularities, difficulty following instructions, orthopedic limitations, women who were pregnant or had reason to believe may become pregnant, persons who had implanted stimulators/electronic devices of any type, and persons with an active infection of any type.

### 2.2. Study Design

**Figure 1:**
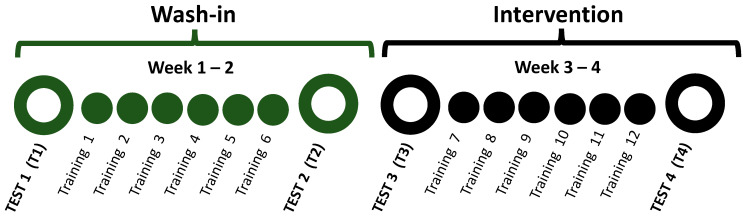
Study Design. The study entailed 4 test days in which ankle angle during a dorsiflexion task, walking, and spasticity measures were collected. The wash-in phase consisted of 2 weeks of locomotor training (Training 1–6), while the intervention phase consisted of either locomotor training combined with transcutaneous spinal or sham stimulation (Training 7–12).

This was a pragmatic clinical trial that used a randomized, wash-in, sham-control design with 4 consecutive weeks of LT. The LT component was directed by physical therapists as part of usual care. Participants were randomized to either an intervention LT + TSS group or a control LT + TSS_Sham_ group. During the first 2 weeks (wash-in phase), all participants received LT 3 days per week. During the second 2 weeks (intervention phase), participants in the LT + TSS group received LT augmented by TSS 3 days per week; participants in the LT + TSS_Sham_ group received LT combined with sham TSS 3 days per week. Outcome assessments for ankle angle during a dorsiflexor activation task, during walking, and during evoked spasticity were conducted at the beginning and end (T1, T2, respectively) of wash-in phase, and at the beginning and end (T3, T4, respectively) of the intervention phase (Figure 1). 

### 2.3. Intervention

#### 2.3.1. Locomotor Training (LT) 

As a pragmatic trial, the LT strategy, as well as rehabilitation outside of the research training, used for each participant was determined by the physical therapist and clinical staff in accordance with standard clinical practice at Shepherd Center. LT ranged from treadmill-based training (with or without body weight support and with or without manual or robotic assistance (Lokomat, Hocoma, Volketswil, Switzerland)) to overground LT (with or without body weight support and with or without manual assistance). 

#### 2.3.2. Transcutaneous Spinal Stimulation (TSS)

Electrical stimulation was applied using a portable, clinical electrotherapy device (Empi Continuum, DJO Global, Vista, CA, USA). The stimulating electrode (5 cm round electrode) was placed over vertebral levels T11/T12 (identified via manual palpation); the reference electrode (10 cm × 15 cm butterfly electrode) was placed over the umbilicus, as this electrode placement has been indicated to stimulate neural inputs to the lumbar spinal cord [28]. For participants randomized to the LT + TSS group, symmetrical, biphasic stimulation with 300 μs pulse width was delivered at 50 Hz. Target stimulation intensity was individualized during each session at an intensity that produced participant-reported paresthesia of the lower legs and feet or to the highest intensity the participant could tolerate. Target intensity was the subthreshold for observable lower extremity muscle activation as indicated by the absence of visible or palpable muscle activation. Stimulation time was standardized to 30 min in duration and was delivered at the target stimulation intensity concomitantly with LT. Following 30 min of stimulation, the intensity was ramped down, the stimulation unit was turned off, the leads were disconnected, and the LT continued as directed. 

#### 2.3.3. Sham-Control Stimulation

The sham-control condition was designed to control for placebo effects associated with the TSS intervention. The stimulator, electrodes, and electrode positions used for the LT + TSS_Sham_ group were the same as described above for the LT + TSS group. Participants were instructed that the stimulation would be reduced to an intensity that they could not feel. The intensity of electrical stimulation was then briefly ramped up to a level at which the participants reported perceiving the stimulation, ramped down to the subsensory level, and then turned off for the remainder of the intervention. The stimulator and electrodes remained attached to the participant for 30 min of the LT session, comparable to the active TSS condition. After 30 min, the stimulator unit was disconnected from the electrode leads. 

### 2.4. Outcome Measures

All measures described below were captured from the more spastic leg as determined by a higher number of ankle clonus oscillations at T1.

#### 2.4.1. Dorsiflexion during Walking

To assess change in swing phase dorsiflexion during walking, we quantified peak ankle angle based on kinematic data captured using inertial measurement unit sensors (XSENS MVN, Xsens Technologies BV, Enschede, The Netherlands). Inertial measurement unit sensors were strapped bilaterally to lower extremities (attached to the pelvis at the center of the back at the height of the anterior superior iliac spine and bilaterally to the middle of the lateral thighs, tibias, and dorsum of the feet inside shoelaces) according to manufacturer guidelines to capture ankle joint angles using inertial motion capture software (XSENS). Calibrated angles were verified during standing with the hip and knee in full extension, and the ankle in the neutral prior to starting the test. Participants walked overground along a 10 m walkway following the instruction to walk as quickly and safely as possible. This test was performed 3 times with a 2 min rest interval between trials. For each walk, the middle 50 percent of steps for the tested foot was analyzed to remove any impact of acceleration and deceleration. Peak sagittal ankle angles at mid-swing were extracted from the sensor data, where positive angles indicated dorsiflexion and negative angles indicated plantarflexion. Based on previous data from treadmill walking in persons with stroke, the minimal detectable change for peak ankle angle during swing phase is 4.9 degrees [29]; this was used as a benchmark in the current study. 

#### 2.4.2. Dorsiflexor Activation Task

To assess the change in ability to activate the dorsiflexor muscles, we assessed both AROM and electromyographic (EMG) activity in the tibialis anterior muscle during a volitional dorsiflexor activation task. Participants were positioned reclined on an adjustable height mat table with both legs extended and shoes removed. Sensor placement was as described above for walking but with shoes off (XSENS). EMG recording electrodes (Motion Lab Systems, Baton Rogue, LA, USA) were placed over the muscle belly of the tibialis anterior midway between the knee and ankle joint. EMG data signals were captured at a 1000 Hz sampling rate using commercial software (Spike CED, Cambridge Electronics Design, Cambridge, UK).

Participants were instructed to pull their toes up toward their head as hard and fast as they could at the onset of a 5 s auditory tone and to maintain that contraction until the tone stopped. This test was performed 3 times with a 30 s rest interval between trials. If background EMG indicated the tibialis anterior muscle was not at rest before or after the tone, or if the dorsiflexion movement elicited ankle clonus, then that trial was discarded, and the test was repeated. EMG data related to the time between the start and end of the auditory tone were processed offline using customized, commercial software (Spike CED, Cambridge Electronics Design, Cambridge, UK) with a root mean square (RMS) filter of 8 ms [8]. AROM and average RMS amplitude of EMG were both analyzed offline using customized MATLAB software (MATLAB, Mathworks, Natick, MA, USA). 

#### 2.4.3. Ankle Clonus Drop Test

The standardized methodology for the ankle clonus drop test was as described previously [27]. The spasticity data analyzed for the current study is a subset of that previously reported [27], and includes data only from those participants who could walk without the use of ankle orthoses with the intent to address questions about the relationship between spasticity and ankle control. Briefly, participants sat upright with back support on the edge of a mat table, with shoes removed and socks left on with inertial measurement sensor (XSENS) placements as described above. The ball of one foot was positioned on the edge of a platform (10 cm height). The mat height was adjusted to ensure that the hip, knee, and ankle joints were at 90-degree angles. The participant’s leg was lifted from beneath the knee until it contacted a T-bar positioned 10 cm above the resting position of the knee. The examiner quickly released the leg allowing the forefoot to impact the edge of the platform. Responses in each ankle were tested 3 times. The number of clonus oscillations during 10 s in each trial was counted offline, and the average number of oscillations for the 3 trials of each test session was used for analysis. Ankle joint oscillations were analyzed offline using customized MATLAB software (MATLAB, Mathworks, Natick, MA, USA). The number of oscillations equating to 4 or greater is indicative of pathological ankle clonus [30].

### 2.5. Data Analysis

Data were analyzed using SPSS (version 26–28; SPSS Inc., Chicago, IL, USA). Descriptive statistics (mean (standard deviation)) of the measures of interest were calculated for each group. Because the small sample precluded the use of interferential statistics for between-group comparisons, and since it has been asserted that effect sizes are more meaningful for clinical interpretation than *p*-values [31], we based comparisons on within-group effect sizes for each measure. Effect size was computed using Cohen’s *d*, and outcomes were categorized as small (0.14), medium (0.31), and large (0.55) effects based on interpretations for rehabilitation data [32]. Spearman’s rank correlations were used to assess associations between measures. To assess the overall effect of 4 weeks of LT on the measures of interest, data were pooled across phases and groups and analyzed using Wilcoxon signed rank tests, and effect sizes were calculated and interpreted as described above. 

## 3. Results

### 3.1. Demographics

Ten participants (including one female) met the inclusion criteria for this study (Table 1). Of these participants, six were randomized to the LT + TSS group and four were randomized to the LT + TSS_Sham_ group. The mean age was 43.67 (20.19) years with an average time of 96 (57.03) days since injury for the LT + TSS group. In the LT + TSS_Sham_ group, the mean age was 36.75 (11.9) years with an average time of 92.5 (22.87) days since injury. For the LT + TSS group, TSS stimulation intensity during the intervention phase had a range of 32–100 mA. 

### 3.2. Dorsiflexion during Walking

Dorsiflexion outcomes related to walking (peak swing phase dorsiflexion angle) and the dorsiflexor activation task (AROM, EMG) for each group can be found in Table 2. 

**Figure 2:**
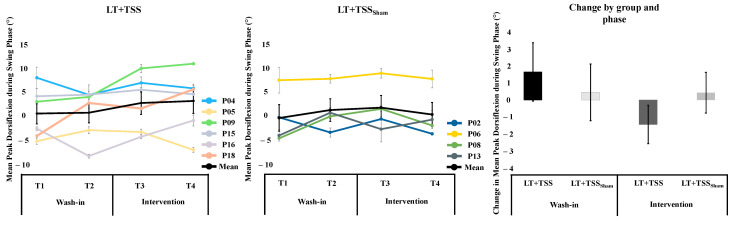
Mean Ankle Angle at Mid-Swing Phase. The average peak ankle angle during swing phase in the more spastic leg in individuals in the LT + TSS group (**left**) and LT + TSS_Sham_ (**center**). Data for individual participants are depicted in color, while the mean is depicted with a bold black line. The change in the ankle angle during the swing phase calculated at the start and end of the wash-in (T1 to T2) and intervention (T3 to T4) phase in each group is depicted in the bar graph (**right**). Error bars represent standard error of mean.

In the LT + TSS group, there was no increase in peak ankle angle during the swing phase in either the wash-in or the intervention phase (effect sizes < 0.14). Conversely, in the LT + TSS_Sham_ group there was increased peak ankle angle during the wash-in phase but a decreased ankle angle during the intervention phase, with medium and small effect sizes, respectively. Only one participant (in the LT + TSS group) had a peak angle increase above 4.9 degrees (P18 during wash-in; Figure 2). 

### 3.3. Dorsiflexor Activation Task

**Figure 3:**
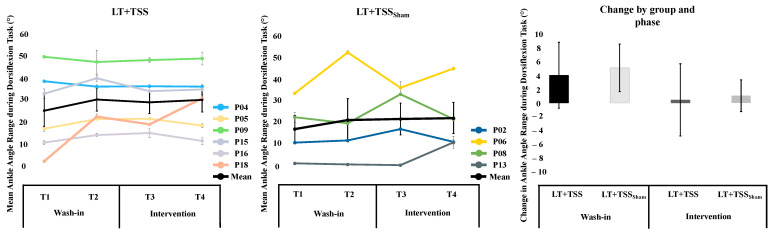
Mean Ankle Angle Range during Dorsiflexion Task. The average ankle angle during the dorsiflexion task in the more spastic leg in individuals in the LT + TSS group (**left**) and LT + TSSSham group (**center**). Data for individual participants are depicted in color, while the mean is depicted with the bold black line. The change in ankle angle during the dorsiflexion task calculated at the start and end of the wash-in (T1 to T2) and intervention (T3 to T4) phase in each group is depicted in the bar graph (**right**). For the bar graph positive values indicate the change was toward more dorsiflexion, and negative values toward more plantarflexion. Error bars represent standard error of mean.

For the dorsiflexor activation task, there was an increase in AROM during the wash-in phase in the LT + TSS group, with a medium effect size. However, there was no effect on AROM observed during the intervention phase. Likewise, in the LT + TSS_Sham_ group, there was increased AROM during the wash-in phase with a small effect size and no change during the intervention phase (Figure 3). 

The LT + TSS group had a decrease in dorsiflexor EMG amplitude during both the wash-in and intervention phases, with small effect sizes. In the LT + TSS_Sham_ group, there was an increase in dorsiflexor EMG during the wash-in phase with a medium effect size and no change during the intervention phase. 

### 3.4. Overall Effects of Locomotor Training on Ankle-Related Outcomes and Relationships among Measures

As equivalent effects were observed in both groups during the intervention phase, suggesting there was no persistent effect of TSS, data were pooled across groups and across study phases (T1–T4) to assess the overall effects of 4 weeks of LT. The average peak ankle angle during the swing phase of gait was 0.33(5.1) degrees at baseline and increased to 2.18 (5.7) after 4 weeks of LT with a medium effect size (*Z* = −0.97, *p =* 0.333, *d* = 0.34). Two individuals from the study had a change in peak ankle angle that exceeded the minimal detectable change of 4.9 degrees (P09: 7.86 degrees; P18: 9.63 degrees). AROM during the dorsiflexor activation task increased with a medium effect size from 22.04(16.40) degrees at baseline to 27.03 (14.22) after 4 weeks of LT (*Z* = −1.48, *p =* 0.139, *d* = 0.33). Active dorsiflexor EMG amplitude was 40.89(30.66) µV at baseline, increasing to 48.13 (26.05) µV at T4 with a small effect size (*Z* = −1.07, *p =* 0.285, *d* = 0.25). The number of ankle clonus oscillations decreased from 11.37 (11.18) beats at T1 to 9.17 (11.17) at T4 with a small effect size (*Z* = −0.28, *p =* 0.778, *d* = −0.2). Additionally, 5 out of 10 individuals (P04: 15.33 average beats; P05: 4.67 average beats; P06: 32 average beats; P13: 24.33; P18: 22.33 average beats) had pathological clonus at T1, but only 3 of those individuals (P06, P13, and P18) remained in the pathological category of clonus at T4 (P04: 3.67 average beats; P05: 2.67 average beats; P06: 32.33 average beats; P13: 8.67; P18: 27.67 average beats). However, in one participant, there was a small increase in the number of beats of clonus that was sufficient to change their classification from not having pathological clonus at T1 (P15: 3.33 average beats) to being categorized as having clonus at T4 (P15: 4.33). 

**Figure 4:**
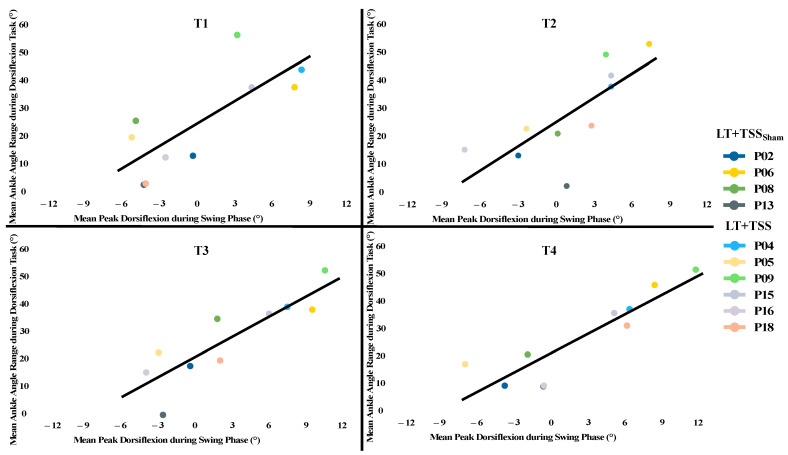
Relationship between peak ankle angle at the mid-swing phase of walking and ankle angle range during the dorsiflexion task. There was a strong significant correlation between the dorsiflexion range of motion in the volitional ankle control task and the peak dorsiflexion angle achieved during the swing phase across all tests.

There were large significant correlations between AROM in the dorsiflexor activation task and peak ankle angle achieved during the swing phase across all timepoints (T1, ρ = 0.64, *p* = 0.024, T2, ρ = 0.82, *p* = 0.002, T3, ρ = 0.87, *p* < 0.001, T4, ρ = 0.82, *p* = 0.002; Figure 4). Additionally, the change (T4–T1) in ankle clonus oscillations had a strong positive association with change in active dorsiflexor EMG amplitude (ρ = 0.78, *p* = 0.004) but not with AROM (ρ = 0.36, *p* = 0.151) or peak ankle angle during swing phase (ρ = 0.19, *p* = 0.305). 

## 4. Discussion

### 4.1. Dorsiflexion during Walking

Due to the importance of dorsiflexor control during the swing phase of walking and the observation of improved walking speed and distance in the LT + TSS group in the larger study of which these data were a subset [27], we predicted that combined LT + TSS would improve dorsiflexion during the swing phase. However, training with combined LT and TSS did not have a persistent impact on dorsiflexion during the swing phase beyond that observed for LT, as there was no effect of an increase in peak ankle angle during the intervention phase in the LT + TSS group. In a prior report, during stimulation, the immediate effects of TSS on the ankle’s range of motion during walking were variable, but three of six participants demonstrated increased ankle range of motion in at least one leg [16]. Given that TSS has been associated with both increased joint range of motion during walking [13] and during volitional tasks [16], and since there is an association between increased foot clearance and increased walking speed [33], it seemed reasonable that the improvements in walking speed and distance with TSS stimulation may be due to an enhanced ability to activate the dorsiflexors. However, our data did not support that hypothesis. 

Based on the findings of these analyses, it seems likely that the increase in walking outcomes exhibited in prior studies may be due to changes in other joints important for foot clearance during walking. For example, in persons with stroke, those with reduced ability to dorsiflex the ankle had an increase in peak hip flexion during the swing phase, resulting in a negative correlation between hip and ankle flexion during the swing phase [34]. Conversely, in an operant conditioning study in persons with SCI, an increase in the tibialis anterior corticospinal tract’s descending drive was associated with increased peak ankle dorsiflexion and improved walking outcomes but not increased knee or hip flexion [25]. However, this kinematic peak joint angle was calculated over the entire step cycle, making it unclear whether the increase was specific to the foot clearance component of the swing phase. Therefore, there is still a possibility of a phase-dependent association between increased hip and knee flexion with decreased ankle dorsiflexion during the swing phase in people with SCI that should be examined in future studies. 

### 4.2. Dorsiflexor Activation Task

In contrast with immediate effects on volitional dorsiflexion observed during TSS [16], training that combined LT and TSS did not have a persistent impact on dorsiflexion AROM. This was indicated by medium and small effect sizes observed in both groups during the wash-in phase, but no effect during the intervention phase for the TSS group (or the TSS_Sham_ group). We predicted that training with 50 Hz TSS would have a persistent effect based on prior evidence that the influence of TSS motor output and spasticity were temporally persistent, for at least 2 h and up to 24 h, respectively, in persons with multiple sclerosis [17]. Considering these findings in the context of prior studies in persons with SCI, it would appear that TSS has an immediate effect on ankle dorsiflexion during the application of the stimulation [16], but these effects do not exhibit temporal persistence. 

### 4.3. Overall Effects of Locomotor Training on Ankle-Related Outcomes

Considering the task-specificity of LT on walking outcomes and the role of central pattern generator circuits in walking, we expected there to be a stronger influence of LT on dorsiflexion during the swing phase than on dorsiflexion during a volitional task. However, we found that the effects of 4 weeks of locomotor training on peak ankle angle during the swing phase of walking and AROM in the dorsiflexor task were equivalent, with an overall medium effect for both. In fact, there was a strong positive correlation between peak ankle angle during the swing phase and AROM during the dorsiflexor activation task. These findings point to the relationship between dorsiflexion during walking, which occurs without conscious volitional effort and has contributions from the spinal central pattern generating circuits [5], and volitional isolated dorsiflexion, which relies on conscious effort to engage direct cortical activation [3,35]. Moreover, this relationship did not change in T4, although some participants received active TSS (Figure 4). There has been recent evidence that corticospinal tract descending drive during a complex ankle control task is associated with walking outcomes [26]. Additionally, although dorsiflexors do not require conscious input during walking, there is evidence that descending supraspinal drive influences walking [24,35]. Therefore, perhaps the degree of corticospinal tract descending drive to the dorsiflexors is what is driving the strong relationship we observed in our study. 

The average peak dorsiflexion angle during the swing phase across participants was around 0 degrees (neutral), which is comparable to a non-injured individual [36]. Some individuals in our study had peak dorsiflexion angles that were above neutral, as has been observed in another study of participants with motor-incomplete SCI [25]. In fact, two participants (P09 and P18) exhibited an increase in peak ankle angle during the swing phase above the minimal detectable change of 4.9 degrees [29]. Although on average the degree change was small, even a small increase in dorsiflexion during the swing phase can enable toe clearance and decrease fall risk. Therefore, there is a need to determine the minimal clinically important change for dorsiflexor activation during walking. As locomotor training is currently the standard of care for individuals with acute/subacute SCI who have the potential to regain walking ability, it is not possible to know whether these improvements would have occurred in the absence of this training in these participants. 

Unexpectedly, after 4 weeks of locomotor training, there was a strong positive association between ankle clonus oscillations, our indicator of reflex responsiveness to the stretching of the plantar flexors, and dorsiflexor EMG amplitudes during volitional activation. Theoretically, increased ability to activate the tibialis anterior activity would be associated with increased reciprocal inhibition to the plantar flexors and therefore decreased reflex activation of the soleus. However, in some persons with SCI, the remodeling of spinal circuits results in a reversal of reciprocal inhibition to reciprocal facilitation [37,38]. Therefore, our results may be due to this phenomenon. 

While we selected 50 Hz TSS because prior work suggests it both improves motor output and decreases spasticity [17], more recent evidence from studies using 30 Hz stimulation suggests this lower frequency may also be effective at influencing our outcomes of interest. Perhaps the effects of TSS in our study may have been more beneficial if 30 Hz was utilized, as this has been shown to have neuromodulatory effects on voluntary locomotor activity [13,16]. However, in a direct comparison of the effects of 30 Hz vs. 50 Hz TSS on locomotor-related outcomes, there was no significant difference between 30 Hz and 50 Hz TSS [16]. 

### 4.4. Limitations

The number of participants who were able to walk without the use of ankle orthotics was small, resulting in a small sample size. In addition, participants’ ability to volitionally activate the dorsiflexors may have not been fully captured as the supine position with knees extended requires the participant to overcome tension in the gastrocnemius. Further, this position may facilitate an extensor response in persons with spasticity. 

## 5. Conclusions

The current study does not provide evidence of a persistent effect of combined TSS and LT on volitional dorsiflexion or dorsiflexion during the swing phase of walking, as there were no differences between the group that did and the group that did not receive TSS. However, after 4 weeks of locomotor training, improvements were observed in all outcome measures. Our findings support the relationship between dorsiflexion during walking and volitional isolated dorsiflexion, which both have shared corticospinal mechanisms, but the latter is influenced by central pattern generators. Factors other than enhanced dorsiflexor activation are likely responsible for improved walking outcomes associated with TSS observed in other studies and should be explored in future studies. 

## Figures and Tables

**Table 1:** Demographics.

Subject ID	Sex	Age (Years)	Time since Injury (Days)	AIS	Neurological Injury Level	LE (Tested)	LEMS (Tested)	LEMS (Total)	Clonus (Tested)	Group
P04	F	53	36	D	C2	L	20	42	15.33	LT + TSS
P05	M	56	84	D	C4	R	20	44	2.67	LT + TSS
P09	M	18	83	D	C7	R	22	33	3.00	LT + TSS
P15	M	54	141	D	C5	R	18	32	3.33	LT + TSS
P16	M	63	185	D	C1	R	22	46	3.00	LT + TSS
P18	M	18	47	D	C5	L	8	33	22.33	LT + TSS
P02	M	43	80	C	C4	R	14	27	2.67	LT + TSS_Sham_
P06	M	37	103	C	C3	L	22	39	32.00	LT + TSS_Sham_
P08	M	47	119	D	C2	L	21	42	3.00	LT + TSS_Sham_
P13	M	20	68	D	C4	R	11	36	24.33	LT + TSS_Sham_

**Abbreviations:** AIS, American Spinal Injury Association Impairment Scale; LE, Lower Extremity; LEMS, Lower Extremity Motor Score; R, Right; L, Left; LT + TSS, locomotor training + active transcutaneous spinal stimulation group; LT + TSS_Sham_, locomotor training + sham transcutaneous spinal stimulation group.

**Table 2:** Outcomes. The measures of interest are displayed above as mean(standard deviation). Effect sizes were calculated using Cohen’s *d*.

	Wash-in Phase				Intervention Phase			
	T1	T2	Difference	Effect Size	T3	T4	Difference	Effect Size
Peak Dorsiflexion during Swing Phase (°)								
LT + TSS	0.76 (5.23)	0.96 (5.21)	0.20 (4.41)	0.04	2.93 (5.67)	3.36 (6.20)	0.43 (2.92)	0.07
LT + TSS_Sham_	−0.31 (5.63)	1.34 (4.75)	1.65 (3.83)	0.32	1.85 (5.12)	0.41 (5.14)	−1.44 (2.52)	−0.28
Ankle Volitional Range during Dorsiflexion Task (°)								
LT + TSS	25.32 (18.41)	30.45 (12.97)	5.13 (8.49)	0.32	29.24 (12.85)	30.30 (13.61)	1.06 (5.68)	0.08
LT + TSS_Sham_	17.12 (13.71)	21.17 (21.64)	4.04 (9.60)	0.22	21.68 (15.99)	22.13 (15.62)	0.45 (10.56)	0.03
EMG during Volitional Task (µv)								
LT + TSS	54.49 (29.73)	49.48 (13.57)	−5.01 (21.01)	−0.22	60.26 (32.59)	52.48 (29.15)	−7.78 (19.75)	−0.25
LT + TSS_Sham_	20.48 (20.53)	31.15 (31.31)	10.67 (14.40)	0.4	43.65 (29.86)	41.60 (24.42)	−2.05 (22.51)	−0.08

## Data Availability

Data will only be made available to users under a data-sharing agreement that provides for (1) a commitment to using the data only for the research purposes identified in the informed consent process and not to identify any individual participant; (2) a commitment to securing the data using good clinical practice data security standards; and (3) a commitment to destroying the data immediately upon authorization following data analysis.

## Clinical Trial Registration

Unique Identifier: NCT03240601.
